# Institutional involvement in the campaign for hand hygiene

**DOI:** 10.1186/1753-6561-5-S6-P122

**Published:** 2011-06-29

**Authors:** MM Baraldi, CZ Talala, MM Simonetti, LB Rezende, CM Santoro

**Affiliations:** 1 Serviço de Controle de Infecção Hospitalar, Hospital Alemão Oswaldo Cruz, São Paulo, Brazil

## Introduction / objectives

The 14th Sanitation Campaign in 2010 had occurred since the planning, execution and finishing with the participation and institutional support. Through the stimulus coming from the oversight and development of a plan of action organized by the of Infection Control Service involving multiple organizational levels, were put into practice the strategies designed to raise awareness of the importance of hand hygiene.

## Methods

This is an experience in aÂ General Hospital, where among the planned activities were encouraged employee participation in the contest for creating a video simulating the technique of hand washing. The disclosure came through the opening event of the annual campaign that brought the video as a reference made by WHO in the same year. By distributing brochures emphasized the regulation considering an interval of three months for delivery of video and awards. Equipment such as video cameras became available and the evaluation of the videos came about through a jury.

## Results

Six videos were entered in the contest, had the participation of 42 employees. The videos were presented to members of the Superintendent, SCIH, Marketing and Research and Education. The criteria were: use of the technique of hand washing, use of image, sound, design and evaluation of the material as a means of institutional promotion. After the presentation and selection of the winning video, awards were extended to all employees who participated.

## Conclusion

Events like this do not replace regular training courses aimed at training of all employees, but call attention to the issue and lead institutional involvement on the basis of a need that is intended to provide safety to the patient against the risk of hospital infection.

## Disclosure of interest

None declared.

